# Development of the Acoustically Evoked Behavioral Response in Larval Plainfin Midshipman Fish, *Porichthys notatus*

**DOI:** 10.1371/journal.pone.0082182

**Published:** 2013-12-10

**Authors:** Peter W. Alderks, Joseph A. Sisneros

**Affiliations:** 1 Department of Psychology, University of Washington, Seattle, Washington, United States of America; 2 Department of Biology, University of Washington, Seattle, Washington, United States of America; 3 Virginia Merrill Bloedel Hearing Research Center, University of Washington, Seattle, Washington, United States of America; University of Maryland, United States of America

## Abstract

The ontogeny of hearing in fishes has become a major interest among bioacoustics researchers studying fish behavior and sensory ecology. Most fish begin to detect acoustic stimuli during the larval stage which can be important for navigation, predator avoidance and settlement, however relatively little is known about the hearing capabilities of larval fishes. We characterized the acoustically evoked behavioral response (AEBR) in the plainfin midshipman fish, *Porichthys notatus*, and used this innate startle-like response to characterize this species' auditory capability during larval development. Age and size of larval midshipman were highly correlated (r^2^ = 0.92). The AEBR was first observed in larvae at 1.4 cm TL. At a size ≥1.8 cm TL, all larvae responded to a broadband stimulus of 154 dB re1 µPa or −15.2 dB re 1 g (z-axis). Lowest AEBR thresholds were 140–150 dB re 1 µPa or −33 to −23 dB re 1 g for frequencies below 225 Hz. Larval fish with size ranges of 1.9–2.4 cm TL had significantly lower best evoked frequencies than the other tested size groups. We also investigated the development of the lateral line organ and its function in mediating the AEBR. The lateral line organ is likely involved in mediating the AEBR but not necessary to evoke the startle-like response. The midshipman auditory and lateral line systems are functional during early development when the larvae are in the nest and the auditory system appears to have similar tuning characteristics throughout all life history stages.

## Introduction

Previous behavioral studies have provided evidence that the auditory system of larval fishes is active during early development and that sound cues may be an important for avoiding predation, navigation, and larval recruitment [1]–[7]. The ability of fish to detect and localize sound during the larval stage may significantly affect mortality and successful recruitment of reef fishes to benthic habitats [8]. Increasing evidence that fish larvae use sound as a navigational cue prior to settlement on reefs has sparked interest in determining the auditory capabilities of larval fishes [9]–[16].

Despite the growing evidence that fishes can detect and localize sound during early development, very little is known about the hearing capabilities of fishes prior to juvenile developmental stages. Behavioral studies have revealed that clownfish (*Amphiprion ephippium* and *A. rubrocinctus*) can respond to sound in embryonic stages just three days post fertilization [17]. In physiological studies that investigated hearing in the zebrafish (*Danio rerio*), Tanimoto et al. [18] demonstrated that auditory responsiveness can occur as early as 40 hours post fertilization while Higgs et al. [19] using the auditory evoked potential (AEP) technique showed that zebrafish of 1.0–4.5 cm total length (TL) had similar auditory tuning profiles. Wright et al. [20]–[22] also used AEPs to investigate the auditory sensitivity of coral reef fish larvae and showed that larvae have hearing abilities similar to that of juvenile reef fish. In contrast, Wright et al. [23] reported ontogenetic and interspecific differences in the hearing abilities of multiple larval fish species with large variations in the auditory capabilities among species tested. While these initial studies are important, more research is needed to determine whether ontogenetic changes in the fish auditory sense correspond to a general pattern of inner ear and auditory central nervous system (CNS) development for all teleost fishes or if the hearing capabilities of larval fishes are species specific and/or environmentally dictated.

The limited seasonal availability of larval fishes combined with their delicate nature make studies of larval fish hearing difficult to conduct. Traditionally, non-invasive behavioral measures that rely on innate responses have allowed researchers to more reliably conduct fish hearing experiments. One such measure, the acoustically evoked behavioral response (AEBR), is well suited for the investigation of hearing in larval fishes. The AEBR is an innate behavioral escape or “startle-like” response that can be evoked by intense acoustic stimuli [24]. In most fishes, the startle response is mediated by large reticulospinal neurons known as Mauthner cells, which activate contralateral spinal motor neurons, and cause the fish to bend in a characteristic “C” shape away from the stimulus source during an escape [25], [26]. Fish lacking or with reduced Mauthner cells exhibit startle-like responses that are less robust without a complete c-start and are often longer in latency than typical Mauthner mediated startle responses [27], [28]. Although startle audiograms may not be as sensitive as other measures, behavioral audiograms based on AEBRs are still a useful non-invasive measure for determining the auditory capabilities of delicate larval fish when other methods can not be used.

Here, we investigate ontogenetic changes in the AEBR in the plainfin midshipman fish (*Porichthys notatus*) as a means to characterize their auditory capability during larval development. The plainfin midshipman has become a neuroethological model for investigating the neural and behavioral mechanisms of audition in teleost fishes [29]–[32]. The focus of this study was to use the AEBR as a measure to determine when the midshipman auditory system becomes functional and whether the lateral line also contributes to acoustic detection during early development. We test the hypothesis that larval midshipman fish are capable of detecting and responding to auditory stimuli during early development, and that larval auditory sensitivity undergoes ontogenetic changes from the early larval to juvenile developmental stages. We interpret our findings as they relate to possible age-related adaptations of the midshipman auditory system for survival during early development.

## Methods

### Ethics Statement

All experimental procedures followed the National Institute of Health guidelines for the care and use of animals and were approved by the University of Washington Institutional Animal Care and Use Committee (protocol 4079-01). Field collection sites in Tamales Bay, California were approved by the California Department of Fish and Game (Scientific Collecting Permit 802021-01, Permanent ID No. SC-4494). Field collection sites at Seal Rock Beach, Washington were approved by the Washington Department of Fish and Wildlife (Washington State Scientific Collection Permit No. 12-192).

### Life History Stage Terminology

The terminology of the various life history stages for embryonic and larval fish is very complex and many different classification systems exist to describe early fish development [33]–[38]. Batrachoidid fishes, including midshipman, lay demersal eggs that undergo development in benthic nests without a pelagic larval stage [39]–[41]. We are unaware of any life history terminology that adequately describes the larval development and parental care of batrachoidid fishes. Therefore, we define the midshipman embryonic stage as the developmental period from fertilization of the ova to when the developing embryos hatch. The larval stage is defined as the time period from hatching to when the larval completely absorb their yolk and detach from the nest substrate. The juvenile stage is defined as the time from when the juveniles become free-swimming after detaching from the nest until they reach sexual maturity. Our description of larval development and terminology is in agreement with what is conventionally defined and used in previous studies for Batrachoidid fishes [41]–[44].

### Animal Collection and Care

We collected rocks with fresh midshipman eggs from the rocky intertidal zone at low tide during the summer breeding season (May- August) from field sites in Tamales Bay, California and at Seal Rock Beach near Brinnon, Washington. Nest rocks with attached eggs were transported back to the laboratory at the University of Washington in coolers with fresh aerated seawater. In the laboratory, the rocks and eggs were placed in 190 L seawater aquaria and kept at 15±2°C. The embryos/larval fish were allowed to develop until they were removed for experimentation.

We monitored and photographed the developing embryos and larvae daily. The photographs allowed us to document when each individual embryo hatched from the egg. We cleaned the embryos/larvae weekly using a small jet of water from a pipet to prevent fungal growth and any sediment build up. Larvae were selected for experimentation based on size. When a larval fish was removed for experimentation, we used a rounded blunt tip knife (approximately 1 cm diameter) to separate the larval fish with yolk from the nest substrate. The fish were then carefully removed using a 5 mL pipet and placed in a glass petri dish with chilled fresh seawater. The larvae and juveniles used in this study ranged in size from 0.6 to 3.3 cm total length (TL). The larval fish were divided into four groups based on TL: small, n = 19, 1.5–1.7 cm TL (1.6 cm TL average); medium, n = 19, 1.8–2.4 cm TL (2.1 cm TL average); large, n = 12, 2.5–2.7 cm TL (2.6 cm TL average); and juveniles, n = 17, 2.8–3.2 cm TL (3.1 cm TL average). These four groups were chosen based on preliminary data to determine if the AEBR thresholds shift during larval development before the lateral line is active (small), after the lateral line develops (medium), just prior to detaching from the natal rock (large), and to compare AEBR thresholds between these larval time points and juvenile fish.

When midshipman larvae were large enough to naturally detach from the rocky nest substrate, they were transferred to a smaller aquarium (3.8 L) with chilled seawater (water temperature was 15±2°C) and quartz sand sediment, which provided substrate for the juveniles to bury themselves during the day. Juveniles were fed a diet of SELCO enriched deshelled live brine shrimp daily.

### Stimulus Calibration

**Figure 1:**
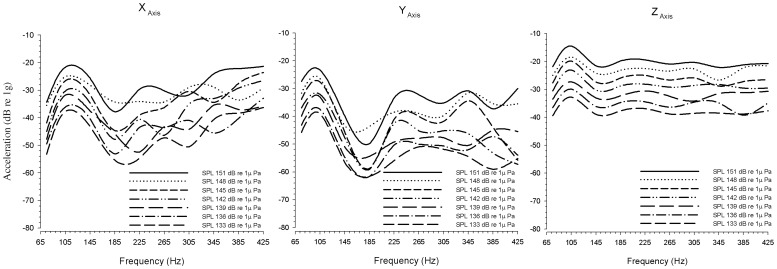
The relationship between particle motion (acceleration) and sound pressure in the experimental tank used to test the AEBR in midshipman fish. Particle motion was measured using a 3D accelerometer after calibrating stimulus frequencies using sound pressure such that all stimulus frequencies at a peak SPL within 2 µPa. Here we display the particle motion measured in the X-, Y-, and Z-axes for all test frequencies and intensities. Note that the Z-axis represents the main axis of stimulation.

Before each experiment the acoustic stimuli were calibrated so that each fish received the same stimulus sound level. Acoustic stimuli produced by an underwater speaker (UW-30, Telex Communication, Burnsville, MN) were calibrated for both sound pressure and particle acceleration. Although *P. notatus* primarily detects the particle motion component of sound, we calibrated our stimulus in terms of sound pressure to allow for a more straightforward comparison between this and previous studies. Furthermore, calibrating stimulus particle acceleration in all three axis (x, y, z) simultaneously can be difficult for an underwater speaker, however calibrating the stimulus produced by the speaker in terms of sound pressure can provide a more consistent measure of the stimulus. We also calibrated the z-axis of particle acceleration (the primary vector of stimulation along the dorsal-ventral axis of the animal) produced by the speaker using an underwater accelerometer (PCB Model 356A32). We verified that both sound pressure levels and the z-axis of particle motion were consistent across all test frequencies. We noted that a 3 dB re 1 µPa change in sound pressure intensity did not translate to a corresponding 3 dB re 1 G change in acceleration in the z-axis of particle motion (see figure 1 for the relationship between sound pressure level and particle motion in these experiments). To measure the particle motion, we played the calibrated stimulus (see procedure below) through the underwater speaker with the 3-axis accelerometer (PCB Model 356A32) 10 cm above the underwater speaker, submerged 5 cm below the surface of the water (occupying the position of the fish). The z-axis was oriented so that it was facing the surface of the speaker in the same position as the dorsal- ventral axis of the fish. The X-axis was oriented in the same position as the rostral- caudal axis of the fish, while the y-axis of the accelerometer was oriented in the same position as the right- left axis of the fish. The accelerometer's output was then amplified (PCB model 482A16) and passed through the analog to digital converter (CED 1401 MKII DAC-ADC) and the particle acceleration calculated using a custom matlab script.

When calibrating the acoustic stimulus to characterize the AEBR, we placed a hydrophone (Bruel and Kjaer 8103) 10 cm above the speaker in the position normally occupied by the fish during an experiment with a constant flow of fresh aerated seawater present just as when conducting the experiments. Stimulus generation was controlled using a custom matlab script. We generated the stimulus using a lock-in amplifier (Stanford Research Systems SR830) that produced the analog signal and passed the signal waveform through an audio amplifier to the underwater speaker. We monitored the amplified hydrophone output on an oscilloscope (Tektronix TDS 2002) and manually adjusted the output level on the audio amplifier until the peak intensity of the signal was 154 dB (±2 dB) re 1 µPa.

Similarly when calibrating the stimulus to test for the sensitivity of the AEBR, the hydrophone was also placed 10 cm above the speaker. We generated the stimulus using a custom matlab script and a digital to analog converter (CED 1401 MKII DAC-ADC) that passed the generated signal though a programmable attenuator (CED 3505) and an audio amplifier to the underwater speaker. The amplified output from the hydrophone was measured and used with the custom Matlab script to automatically compensate for differences in sound intensity at the test frequencies. The calibration script adjusted the output voltage for each test frequency so that the sound pressure was of equal amplitude within ±2 dB re 1 µPa. We then verified the speaker calibration by measuring the stimulus frequencies relative to each other using a spectrum analyzer (Stanford Research Systems SR780). During the experiment, we made adjustments to the sound intensity level by adjusting the programmable attenuator in 3 dB re 1 µPa steps (CED 3505).

### Characterization and Onset of the Acoustically Evoked Behavioral Response

In order to characterize the AEBR and determine the size/age larvae begin to exhibit the AEBR, we detached 62 midshipman larvae from their natal rock and individually glued their external yolk to an acrylic disk (1.5 cm diameter, 0.75 cm thick) using cyanoacrylate glue. Thirty-eight of the 62 animals used in these experiments were of known post hatch age ranging in size from 0.6 to 3.3 (mean size  = 1.46±.58) cm TL and age from 1 to 47 (mean age  = 20.87±12.3) days post hatch (Methods S1, Figure S1, Results S1). After the yolk was attached and the animal was ready to be tested, the disk was submerged and positioned 5 cm under the surface of the water and 10 cm above the underwater speaker in a cylindrical Nalgene tank (30 cm diameter, 24 cm high) that was resting on a vibration isolation table. The water temperature of the tank was maintained at 15±2°C and the fish were provided with chilled aerated seawater throughout the experiment. The acrylic disk holding the fish was suspended above the speaker using an acrylic support structure that was attached to the vibration isolation table. Because midshipman are nocturnal, we performed all of the experiments in a darkened sound attenuation booth. Fish were given 5 min. to acclimatize to the water and recover from handling before any experiments were initiated. All experiments were video recorded for later analysis using a low light camera with a video capture rate of 30 frames/second.

**Figure 2:**
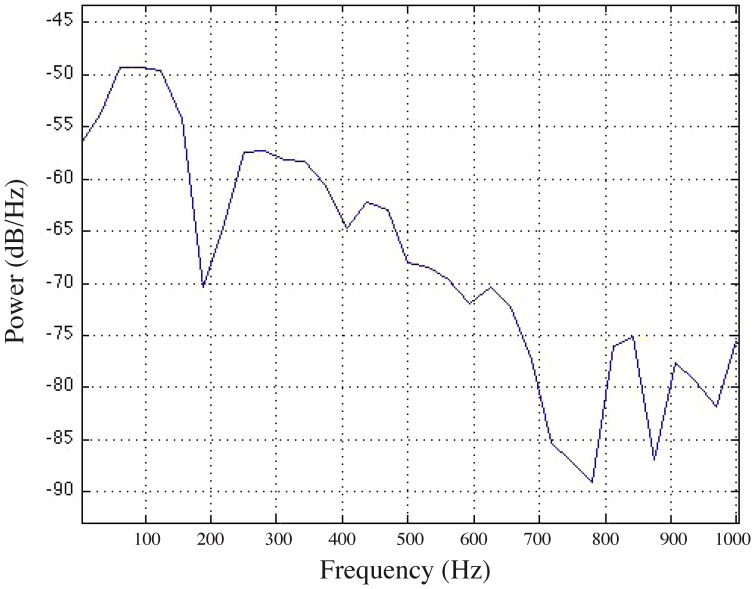
The power spectrum of the complex click stimulus used to identify the size/age for the onset of the acoustically evoked behavioral response. The majority of the energy in the stimulus is located below 700 µPa or −15.2 dB re 1 g in the Z-axis of stimulation. Juvenile and adult midshipman have greatest auditory sensitivity at frequencies below 300 Hz.

**Figure 3:**
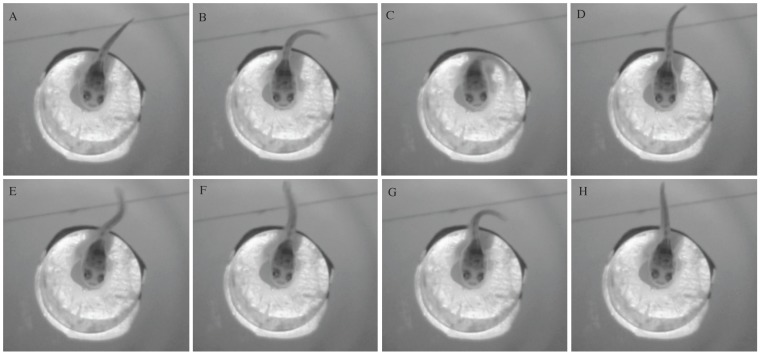
Video frame sequence of a representative acoustically evoked behavioral response (AEBR). The images show a 2.1–H show the fish positions during and after stimulus presentation. Note that in image C the caudal fin is curved toward the head of the fish, almost forming a C shape. A positive AEBR was only considered when the caudal fin moved greater than ½ of the fish's total length directly following a stimulus presentation.

We presented the fish with complex, broad-band, click stimulus that had a peak amplitude of 154 dB (±2 dB) re 1 µPa (−15.2 dB re 1 G z-axis) 3 times per trial using a custom matlab script. A subset of the animals (n = 12) received stimulus presentations with either a 30 second, 1 minute, 2 minute or 5 minute inter-stimulus interval. Having multiple presentations of the same stimulus allowed us to determine the optimal inter-stimulus interval that we later used to test the frequency sensitivity of the AEBR. We determined that a 2-minute inter-stimulus interval was the optimal interval that prevented stimulus habituation. The complex click stimulus was chosen because it was broadband and contained a high concentration of energy at frequencies below 200 Hz (Figure 2). Similar to other fishes that do not have specialized adaptations for hearing high frequencies, the plainfin midshipman is most sensitive to frequencies below 200 Hz [32]. The midshipman AEBR consisted of quick posterior thrust of the pectoral fins followed by rapid undulation of the caudal fin (Figure 3). Because some movement of the pectoral fins and caudal fin is associated with opercular movement and normal ventilation of the gills, a positive AEBR was only considered when the caudal fin moved greater than 50% the fish's total length directly following a stimulus presentation. This response criteria represents a conservative estimate as such large movements were only witnessed in response to intense acoustic stimuli or when fish were physically handled. Undulations of the caudal and pectoral fins during an AEBR commonly lasted several seconds, but had durations up to 45 seconds. The caudal undulation component was the most reliable measure of the AEBR to intense acoustic stimuli. We were unable to measure the latency of the AEBR due to the lack of a high-speed camera. Other measures of response, such as ventilation rate, were not used because it was difficult to observe the opercular movements under low light conditions.

### Frequency Sensitivity of the Acoustically Evoked Behavioral Response

We tested the sensitivity of the AEBR to pure tone stimuli in order to determine how the auditory system of larval midshipman responds to tonal acoustic stimuli, which is similar to the tonal components of the advertisement call that is produced by the male while in the nest. We adapted the experimental setup used previously to determine the developmental onset of the AEBR. The only difference in these experiments was that we used a parafilm support to suspend the larvae above the underwater speaker instead of the acrylic support structure. Small and medium midshipman larvae were glued (cyanoacrylate glue) directly to the parafilm support via their external yolk, whereas the large larvae and juveniles were placed in a parafilm cup positioned on the parafilm support suspended above the underwater speaker. After gluing the larvae to the parafilm support, fish were given a 5-minute acclimatization period that allowed them to recover from handling. It was not possible to glue the large larvae or the juvenile fish to the parafilm support due to the lack of an external yolk. The parafilm support provided greater acoustic transparency than other alternatives that allowed fish to be maintained 10 cm above the underwater speaker. Fish were supplied with continuous flow of chilled, aerated seawater that was maintained at 15±2°C. The experiments were performed in a darkened sound attenuation booth and videotaped for later analysis.

All fish were randomly presented 100 ms pure tone stimuli with a 6 ms ramp at 75 Hz and 105 to 425 Hz with 40 Hz increments. Each stimulus presentation was followed by a 2-minute inter-stimulus interval. We varied intensities from 154 to 136 dB re 1 µPa always beginning at 154 dB re 1 µPa and decreasing in 3 dB steps. We used the same response criteria that were used to characterize the AEBR (i.e. undulations of the caudal fin greater than ½ TL) and determine the AEBR thresholds. We then compared the overall AEBR threshold profiles for the four groups of fish (small, medium and large larvae and juveniles).

### Lateral Line Visualization

In order to determine if the mechanosensory lateral line is involved in mediating the AEBR, we first visualized the distribution and number of neuromasts using the vital dye DASPEI (2-[4-(dimethylamino)styryl]-N-ethylpyri diniumiodide, Invitrogen Molecular Probes, Eugene, OR) similar to Harris et al. [45]. Thirty-five midshipman larval were immersed in a 0.005% concentration of DASPEI and chilled seawater for 15 minutes and then rinsed twice in chilled seawater. We then anesthetized the fish using 1.6 mL MS-222 (3-aminobenzoic acid ethyl ester, methansulfoneate salt, Sigma-Aldrich, St. Louis, Missouri) and visualized the active neuromast cells *in vivo* using a fluorescent dissecting microscope (Leica MZ12 FL111, Leica Microsystems GMBH; DASPEI filter set (excitation 450–490 nM and barrier 515 nM; Chroma Technologies, Brattleboro, VT).

To verify the DASPEI results, we used phalloidin (Alexa Fluor 488 phalloidin, diluted 1∶100; Invitrogen) to label the neuromast hair cells [46]. Fish were euthanized by overdose of MS-222. We removed patches of epidermal tissue from the operculum, anterior trunk, and dorsal cranium from the euthanized fish and fixed overnight in 5% paraformaldehyde. These patches were chosen because they represent areas where the anterior and posterior lateral line develops earliest [47]. The tissue was then rinsed in 1xPBS and stained using 0.001% phalloidin for 20 min. followed by two rinses of 1xPBS. We visualized the tissue using a fluorescent light microscopy (Leica DMR microscope, Leica Microsystems GMBH; GFP Filter Set) at 40x. This data was used to help interpret the AEBR results.

### Statistical Methods

Both AEBR onset and DASPEI staining data were analyzed using non-linear regression to find the best-fit model (a numerical optimization algorithm was applied in SigmaPlot software, Systat Software Inc., to determine the best-fit parameters using a least squares fitting technique that minimized the global minimum of a sum of squares for the data). Best frequency sensitivity data of the AEBR was analyzed using one-way ANOVA followed by a Bonferroni post-hoc analysis to analyze any differences. Frequency sensitivity data were analyzed using a multivariate analysis of variance MANOVA (AEBR threshold was the dependent variable and frequency and size class were the fixed factors). Because we are primarily interested in differences between the four size classes at each stimulus frequency, the MANOVA analysis was followed by an *a priori* ANOVA analysis at each stimulus frequency and Bonferroni post-hoc analysis to analyze differences between the four size groups. SPSS statistical software was used to perform all statistical analyses.

## Results

### Onset and characterization of the acoustically evoked startle-like response

**Figure 4:**
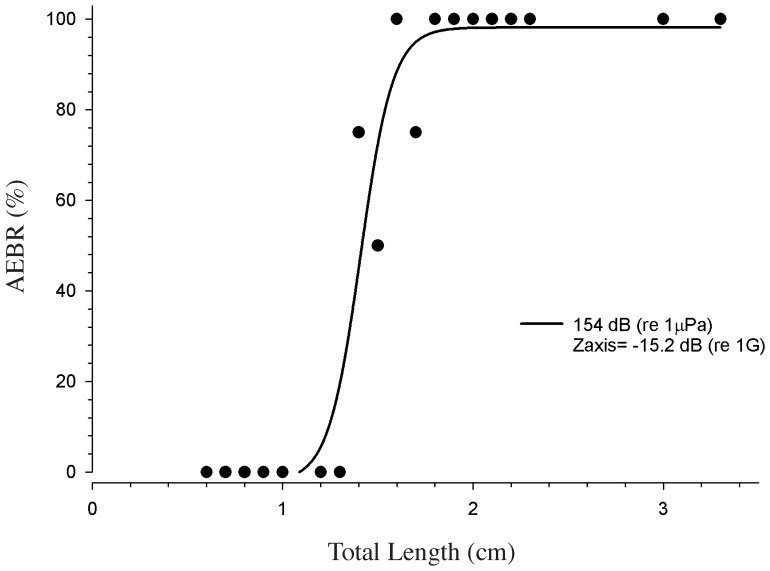
The acoustically evoked behavioral response (AEBR) of fish to a complex click stimulus with a peak SPL of 154 dB re 1 µPa or −15.2 dB re 1 g in the Z-axis of stimulation. The AEBRs are shown as the percentage of the tested fish (60 midshipman larvae and 2 juveniles) that responded to the stimulus. Note that none of the small midshipman larvae less than 1.4 cm TL responded to the stimulus, whereas all of the midshipman larvae greater than 1.8 cm TL responded. Thus onset of the acoustically evoked behavioral response is estimated to occur between 1.4–1.8 cm TL. The solid line represents a best-fit sigmoidal curve.

The duration of the caudal fin undulations ranged from 1.5 to 45 seconds with an mean duration of 4.3±3.5 SD seconds. Following the initial vigorous undulations of the caudal fin, the fish would then stop all body movement with the exception of the respiratory movements of the operculum and small spontaneous movements of the pectoral and caudal fins. A sigmoidal function (i.e., a 4 parameter sigmoid with the equation: f = y0+a/(1+exp(−(x−x0)/b)), r^2^ = 0.94, K- S Statistic = 0.337) provided the best fit for the relationship of the AEBR with TL. A response rate of 50% for the AEBR corresponded to a fish size of 1.5 cm TL (Figure 4). We did not observe AEBRs in fish smaller than 1.4 cm TL, and all fish (100%) greater than 1.8 cm TL responded to the intense acoustic startle stimuli. Our data suggest that the AEBR can first be evoked at a size of 1.4 cm TL or an age of 23 days post-hatch development (Figure S1, Results S1).

### Frequency sensitivity of the AEBR

**Figure 5:**
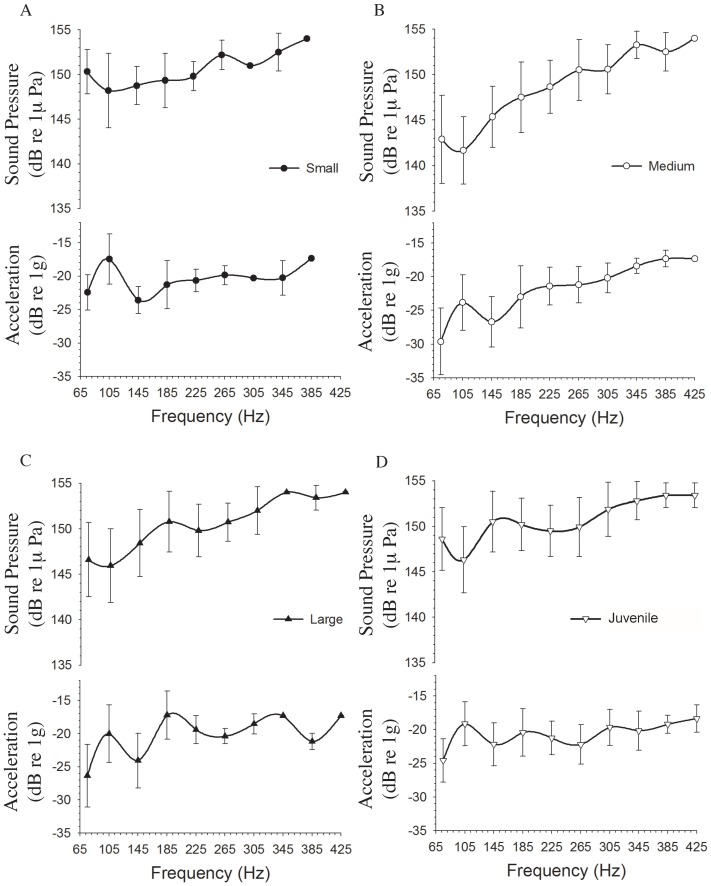
Acoustically evoked behavioral response (AEBR) profiles for the four size groups of midshipman larvae (small, medium, large) and juveniles. The top portion of the graphs shows the response profiles in terms of SPL and the bottom portion of each graph is displayed in terms of acceleration (particle motion) in the Z (vertical)-axis of stimulation. Small midshipman larvae (A) are depicted by the line with solid circles, medium midshipman larvae (B) with open circles, large midshipman larvae (C) with solid triangles, and the juveniles (D) with open triangles. Over all the response profiles for all four groups were similar in shape with greatest sensitivity at the lowest test frequencies (<225 Hz).

**Figure 6:**
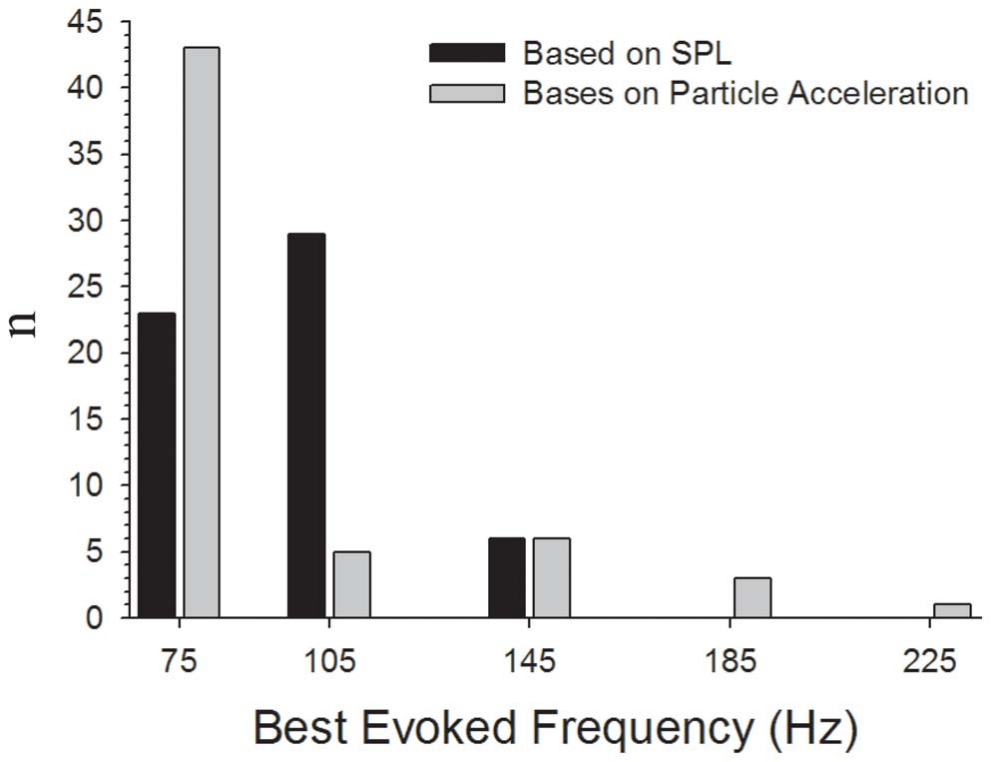
Best evoked frequency (BEF) histograms of the acoustically evoked behavioral response in midshipman larvae based on sound pressure level (SPL, black bars) and particle acceleration (gray bars). The distribution of the BEF for the AEBR is based on the individual AEBR profiles for all the midshipman larval groups tested. Note that the BEF is defined as the frequency with the lowest threshold to evoke the AEBR).

In general, thresholds for the AEBR were lowest at frequencies below 145 Hz and the sensitivity to tonal stimuli gradually decreased at higher frequencies gradually with highest thresholds found at the highest frequency that evoked a behavioral response (Figure 5). Overall significant effects were found for frequency (MANOVA F_9, 62_ = 32.94, p<0.001) and developmental group (MANOVA F_3, 62_ = 6.41, p = 0.001) as well as a significant frequency * developmental group interaction (MANOVA F_27, 62_ = 4.13, p<0.001). Significant threshold differences between size groups for the AEBR were observed at 75 Hz (ANOVA F_3, 59_ = 10.37, p<0.001), 105 (ANOVA F_3, 61_ = 8.1, p<0.001), and 145 Hz (ANOVA F_3, 52_ = 6.73, p = 0.001). Post-hoc analysis revealed that at 75 Hz the medium larvae had lower AEBR thresholds than that of small larvae (Bonferroni, mean difference  = 7.4 dB, p<0.001) and the juveniles (Bonferroni, mean difference  = 5.7 dB, p<0.001). Medium larvae also had lower AEBR thresholds than that of small larvae (Bonferroni, mean difference  = 6.5 dB, p<0.001), large larvae (Bonferroni, mean difference = 3.9 dB, p = 0.031), and juveniles (Bonferroni, mean difference  = 4.3 dB, p = 0.013) at 105 Hz. At 145 Hz the medium larvae had lower AEBR thresholds than the large larvae (Bonferroni, mean difference  = 3.2 dB, p = 0.041) and juveniles (Bonferroni, mean difference  = 5.2 dB, p<0.001). No other differences in AEBR thresholds among the four test groups were observed. Best evoked frequencies (BEF, defined as the frequency with the lowest threshold for the AEBR) ranged from 75 to 145 Hz (Figure. 6). The AEBR threshold at BEF ranged from 133 to 151 dB re 1 µPa or −32 to −14 dB_z-axis_ re 1 G. The distribution of BEFs for the AEBR did not differ across size class (one-way ANOVA, F_3, 57_ = 2.24, p = 0.095), however, the medium larvae (1.9–2.4 cm TL) did have significantly lower AEBR thresholds at BEF then all other size groups (one-way ANOVA, F_3, 57_ = 7.64, p<0.001; Bonferroni post-hoc comparisons: medium vs. small: mean difference  = 6.7 dB, p<0.001, medium vs. large: mean difference  = 4.8 dB, p = 0.026, medium vs. juveniles: mean difference  = 4.8 dB, p = 0.014). No other differences in AEBR threshold at BEF were observed between the size classes.

### Development of the lateral line

**Figure 7:**
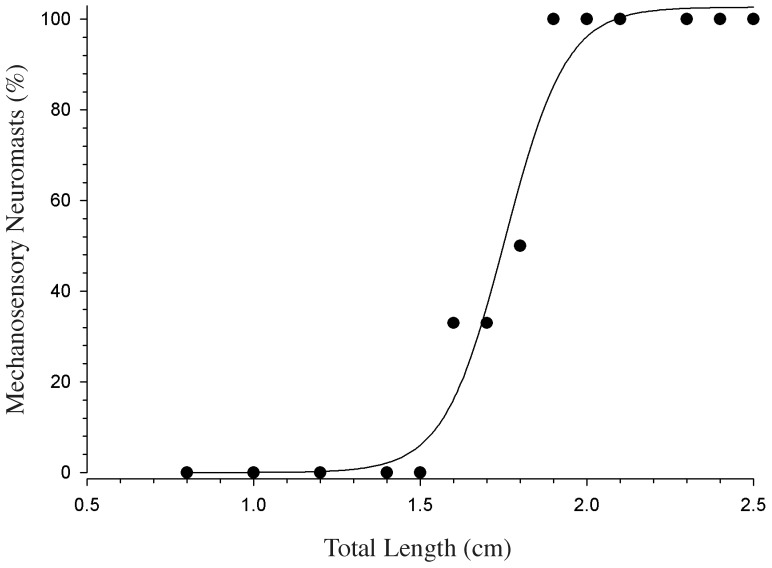
The presence of mechanosensory neuromasts as a function of fish total length (TL) in midshipman larvae. Mechanosensory neuromasts are shown as the percentage of fish examined that had neuromasts present. The presence of mechanosensory neuromasts was determined by the uptake of the vital dye DASPEI, which is taken up by energetically active cells such as lateral line neuromasts and can be visualized *in vivo*. Fish were scored base on DASPEI staining in a binary fashion (yes/no): yes, for the staining of one or more neuromasts and no, for a lack of neuromast cell staining. Note that none of the small midshipman larvae less than 1.6 cm TL had any detectable neuromast cells, whereas all larvae greater than 1.8 cm TL had at least one neuromast cell with DASPEI staining. The solid line represents a best-fit sigmoidal curve.

The percentage of fish having neuromasts at a given size is shown in Figure 7 and the relationship of neuromast DASPEI staining of the lateral line and body size (TL) was best fit according to a sigmoidal function (i.e., a 3 parameter sigmoid with the equation: f = a/(1+exp(−(x−x0)/b), r^2^ = 0.98, K- S Statistic = 0.286). Fish less than 1.6 cm TL were not observed to have any lateral line neuromasts; however 50% of the fish at a size of 1.8 cm TL had superficial or canal neuromasts. All fish greater than 1.9 cm TL had at least one superficial or canal neuromast present. The negative DASPEI staining results were verified using a post fix phalloidin fluorescent stain. Our results show that lateral line neuromasts are first observed in larval fish at a size of 1.6 cm TL, which corresponds to fish >27 days old post hatch (Figure S1, Results S1).

## Discussion

### The acoustic evoked behavioral response

The AEBR is an innate startle-like response that can be evoked by intense acoustic stimuli [24]. Startle responses can be elicited by acoustic, visual, or tactile stimuli and serve the adaptive function of initiating escape responses [25], [48]–[51]. Escape behaviors are evolutionarily conserved due to their survival value in, but not limited to, predator-prey interactions [52], [53]. In fishes, the acoustic startle response is mediated by relatively large brainstem reticulospinal neurons (RSNs) called Mauthner cells that receive information from ipsilateral sensory afferents and synapse with contralateral spinal motor neurons [26], [54], [55]. When activated, the Mauthner cells depolarize and cause the contralateral motor neurons to fire synchronously and the fish bends into a characteristic “C” shape away from the stimulus source. The development and evolution of the c-start startle response has been studied in many fish species [56]–[58]. The innate c-start startle behavior has also been exploited to better understand fish hearing [58], [59] and to determine the onset of hearing in larval fishes [24], [60]. However, not all fish have Mauthner cells. Fish without Mauthner cells exhibit startle-like responses that are often substantially longer in latency and lack the characteristic “C” shape body bend [27], [28]. Removal of the Mauthner neurons results in escape behaviors that have similar characteristics to behavioral responses in fishes without Mauthner cells [28], [61], [62]. This non-Mauthner escape pathway is mediated by the MiD3cm RSN. [63]–[67]. Plainfin midshipman fish do not have Mauthner cells but do exhibit a longer latency AEBR. Both the acoustic startle and AEBR likely serve a similar function in the initiation of escape behaviors; however further research is necessary to determine if AEBRs are mediated via the MiD3cm neuron or analogous RSN or via a different reticulospinal hindbrain pathway in the plainfin midshipman.

### Early ontogeny and auditory development

In this study, we show that the AEBR can be used as a conservative measure to determine the age at which *P. notatus* first begins to respond to sound. Research on other fishes has shown that startle responses can be acoustically evoked when the auditory end organs and their innervation are developed [60]. The auditory system likely first becomes active in larval midshipman between 1.4 and 1.8 cm TL (our small group for the AEBR threshold experiments). Using 50% AEBR response rate as our benchmark, midshipman larvae begin hearing at 1.5 cm TL. Future work will be necessary to determine the relationship between the onset of hearing and the connectivity and development of the peripheral and central auditory systems in larval midshipman fish.

### AEBR thresholds

The AEBR is an innate response evoked by intense acoustic stimuli similar to an acoustic startle response [68]. The resulting behavioral thresholds and response profiles of the AEBRs represent a conservative measure of auditory sensitivity [69]. Although the thresholds for the AEBRs may not represent absolute hearing sensitivity, they are a useful measure of hearing when working with very delicate animals where other techniques may be too invasive.

The overall shape of the AEBR profiles revealed lowest thresholds at frequencies below 225 Hz and response sensitivity that gradually decreased at higher frequencies in all size groups tested. Auditory tuning profiles based on saccular potential and single unit recordings in juvenile and adult plainfin midshipman are similar in shape to that of the AEBR profiles reported here [32], [70]. Sensitivity to low frequency sound appears to be conserved throughout all life history stages from early larval to adult fish in *P. notatus*. The sensitivity of the midshipman auditory system to low frequencies (<225 Hz) can partially be explained by structure of the inner ear. The saccule, the primary auditory end organ in the midshipman and most teleosts, is an otolithic organ that responds to the particle motion component of sound much like an accelerometer [71]–[73]. The saccule contains a dense otolith, which moves at a different phase and amplitude from the saccular epithelium as sound passes through the ear [74]–[76]. As the otolith and sensory epithelium move past each other, the hair bundles bend generating a receptor potential [77]–[80]. This system is inherently most effective at responding to low frequencies [81], [82]. Another possible explanation for enhanced hearing of low frequencies in plainfin midshipman is a co-evolution of the vocal and auditory systems to enhance communication. Adult midshipman use specialized sonic muscles to vibrate their swim bladder to produce vocalizations [83]–[85]. The sonic muscles contract at the same rate as the fundamental frequency of the midshipman calls. As a result, the majority of the energy in midshipman vocalizations is concentrated in low frequencies [29], [86], [87].

The medium-size midshipman larvae had significantly lower AEBR thresholds at BEFs as well as significantly lower AEBR thresholds at 75, 105, and 145 Hz test frequencies. There were not significant differences between any of the other size groups. The greatest mean difference between the medium and large larvae was at 75 Hz, which was only 4.7 dB. This result is significant, however it is a relatively small difference and likely does not reflect a behaviorally relevant difference in auditory sensitivity. One possible explanation for this difference is that inner ear hair cells are being added at a greater rate during the larval stage than in other life history stages [88]–[90]. During early life history stages, dendritic arborization and ganglion cell numbers increase rapidly [91]. It is possible that in medium-sized midshipman larvae hair cell addition briefly outpaces ganglion cell addition such that the convergence ratio increases at a greater rate than in other larval stages. This possible increase in convergence may temporarily result in greater AEBR sensitivity. However, further research is necessary to determine the mechanisms responsible for these differences in AEBR sensitivities between these size groups of midshipman larvae.

### Role of the lateral line system in mediating AEBRs

The lateral line system is composed of mechanoreceptive organs known as neuromasts that detect information about hydrodynamic flows over the skin in fishes [92]–[97]. Adult plainfin midshipman have both canal neuromasts (located primarily on the head and operculum) and superficial neuromasts located on the head and four rows descending caudally along each side of the body trunk [98]. The lateral line system develops soon after the AEBR is first observed in small midshipman larvae. Using the *in vivo* DASPEI and postfix phalloidin stains, we were unable to visualize the presence of any lateral line neuromasts in fish smaller than 1.6 cm TL while at a size of 1.8 cm TL only 50% of the examined fish had superficial neuromasts. The lateral line system did not appear fully developed until fish were greater than 2.0 cm TL.

It is likely that the detection of acoustic stimuli by the lateral line influences the AEBR, but our data suggests that lateral line input is not necessary to evoke the AEBR since some larvae (n = 3) without superficial or canal neuromasts exhibited AEBRs. However, both the superficial and canal neuromasts of the lateral line may be involved in mediating the startle-like behavior later in development since the inner ear and lateral line organ are thought to have overlapping receptive fields [99]. Recently in the goldfish (*Carassius auratus*) the lateral line has been demonstrated to be involved in fish hearing and the encoding of directional information as well as having an effect the onset latency of the escape response [100], [101]. It is possible that the onset latency of the AEBR decreases as the lateral line becomes more developed in *P. notatus*, however future research is necessary to determine if such an ontogenetic relationship exists. We should note that the medium-sized midshipman larvae (1.8–2.4 cm TL), which were the most sensitivity to the startle stimuli, were at the size when the superficial and canal neuromasts first appear and undergo significant proliferation. The medium sized larvae had significantly lower BEF sensitivities than both the small and large larvae. Future work should focus on AEBR sensitivity and development of the medium size larvae to determine the mechanisms for this apparent increase in hearing sensitivity.

### Hearing in juvenile plainfin midshipman

While direct comparisons of results obtained by physiology and behavioral methods are difficult at best, the present data represent a unique opportunity to compare the results obtained by AEBR and saccular potential recordings since juvenile midshipman of overlapping size were used in both studies. The present study used a similar experimental setup as the author's previous ontogenetic study in plainfin midshipman [32]. Overall the response profiles of the juvenile midshipman have a similar shape with greatest sensitivity at low frequencies with response sensitivity gradually decreasing at higher frequencies. However, the slopes of the profiles differ with the response profiles generated from the saccular potential recording technique having a steeper slope than profiles generated from AEBRs (*i.e.* there are greater differences in mean thresholds at lower frequencies than higher frequencies). The response profile from the saccular potential recordings was also on average 17 dB re 1 µPa more sensitive than profiles generated by AEBRs. The frequency with the lowest response threshold in both studies ranged from 75 to 145 Hz for juvenile fish. However the threshold at BEF (best frequency in Alderks and Sisneros [32]) was on average 26 dB re 1 µPa lower using the saccular potential recording technique. AEBRs require an intense acoustic stimulus; therefore they have much higher thresholds than absolute hearing thresholds. The main benefit to using AEBRs to test hearing is that the innate response requires no conditioning and the non-invasive nature of the technique allows it to be used on delicate larval fish.

## Supporting Information

Figure S1 **The relationship between size and post-hatch age of 38 midshipman larvae.** Post-hatch age data of larva fish were recorded from 6 different nests and the age was then correlated with TL. Size and post-hatch age were highly correlated (r^2^ = 0.92, p<0.001).
(TIF)

Methods S1 **Post hatch larval growth analysis.**
(DOCX)

Results S1 Larval growth analysis.
(DOCX)
