# Supplementary figures and images for: Development of the Acoustically Evoked Behavioral Response in Larval Plainfin Midshipman Fish, Porichthys notatus
Source: PLoS One. 2013 Dec 10;8(12):e82182. doi: 10.1371/journal.pone.0082182 (PMC3858275; doi:10.1371/journal.pone.0082182)

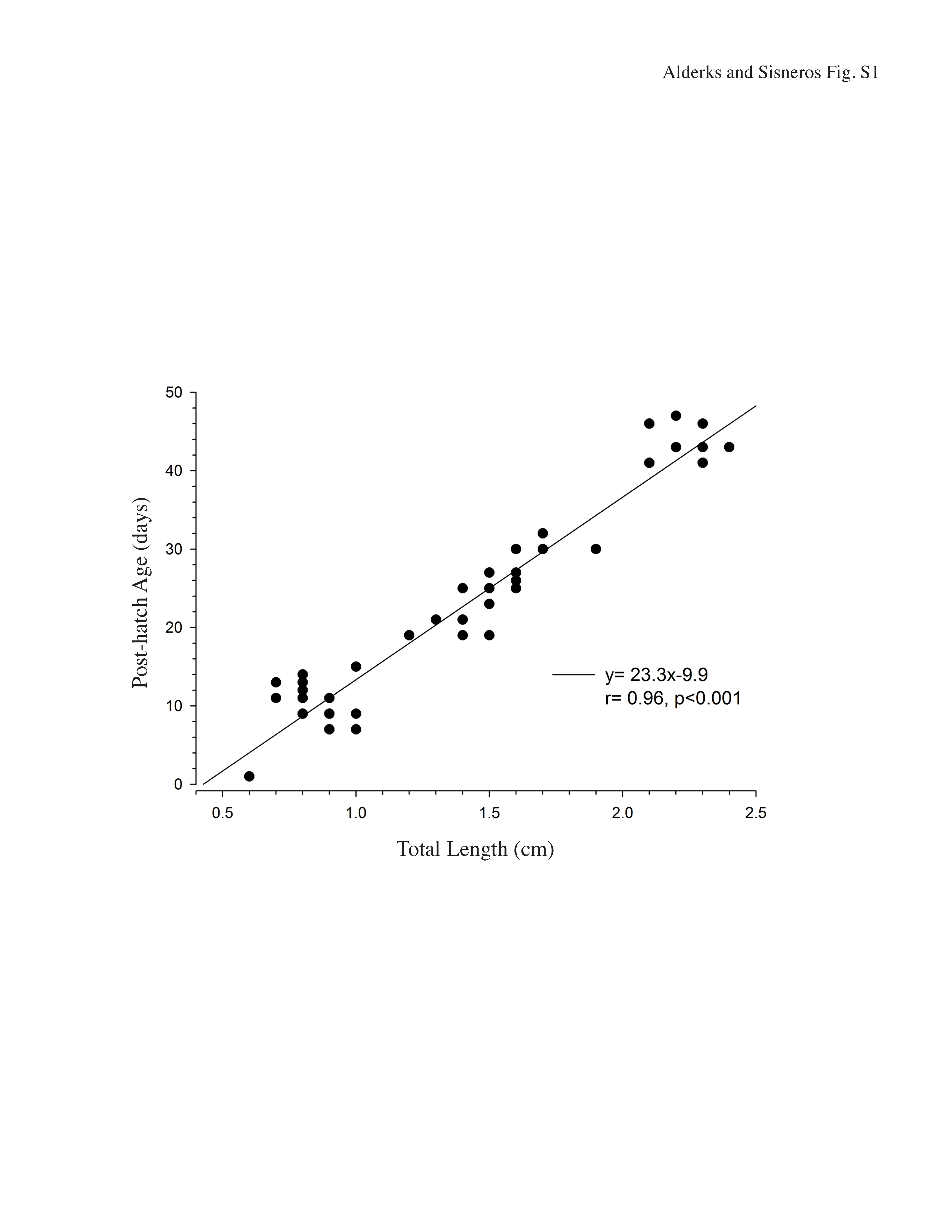

Supplement: Figure S1 — The relationship between size and post-hatch age of 38 midshipman larvae. Post-hatch age data of larva fish were recorded from 6 different nests and the age was then correlated with TL. Size and post-hatch age were highly correlated (r2 = 0.92, p<0.001). (TIF) [file pone.0082182.s001.tif]
