# Supplementary material for: Development of the Acoustically Evoked Behavioral Response in Larval Plainfin Midshipman Fish, Porichthys notatus
Source: PLoS One. 2013 Dec 10;8(12):e82182. doi: 10.1371/journal.pone.0082182 (PMC3858275; doi:10.1371/journal.pone.0082182)
Supplement: Methods S1 — Post hatch larval growth analysis. (DOCX) [file pone.0082182.s002.docx]

**Methods S1**

*Post hatch larval growth analysis*

We investigated the relationship between size and age in the plainfin midshipman. Because midshipman are difficult to breed in the lab, we chose to quantify post-hatch development and determine larval age from the date that embryos hatched from eggs; nests containing recently spawned eggs were collected from the field and incubated in the lab. Although age post-hatch does not give absolute age, it does give a useful reference time point of development and the age relative to hatching date. Because growth rates of developing fish are known to be temperature dependent [102-104], we maintained the embryos and larva at a relatively constant temperature of 15 °C ± 2 °C, which is similar to summer water temperatures in the intertidal region at our field collection sites. In order to determine post-hatch age and development, we collected six large nest rocks with fresh eggs attached and brought them into the lab where we could monitor them daily in a controlled environment. These nests were photographed and individual eggs/embryos were noted and given a number so we could individually monitor the 38 developing embryos/larvae over the course of incubation. We noted the date when individual embryos hatched (day = 0) and measured the TL of the larvae after removal for experimentation. Growth data were analyzed using a linear regression to determine the relationship between body length and post hatch age.

**References**

102. Villamizar N, Ribas L, Piferrer F, Vera LM, Sanchez-Vazquez JF (2012) Impact of Daily Thermocycles on Hatching Rhythms, Larval Performance and Sex Differentiation of Zebrafish. Plos One 7: e52153. DOI:10.1371/journal.pone.0052153.

103. O'Brien TP, Taylor WW, Briggs AS, Roseman EF (2012) Influence of water temperature on rainbow smelt spawning and early life history dynamics in St. Martin Bay, Lake Huron. J Great Lakes Res 38: 776-785.

104. Grasman J, van Deventer WBE, van Laar V (2012) Estimation of parameters in a bertalanffy type of temperature dependent growth model using data on juvenile stone loach (*Barbatula barbatula*). Acta Biotheoretica 60: 393-405.
