# Supplementary material for: Development of the Acoustically Evoked Behavioral Response in Larval Plainfin Midshipman Fish, Porichthys notatus
Source: PLoS One. 2013 Dec 10;8(12):e82182. doi: 10.1371/journal.pone.0082182 (PMC3858275; doi:10.1371/journal.pone.0082182)
Supplement: Results S1 — Larval growth analysis. (DOCX) [file pone.0082182.s003.docx]

**Results S1**

*Larval growth analysis*

Larvae hatched at a size range of 0.5 to 0.6 cm TL (mean = 0.54 ± 0.05 cm SD) and detached from the nest at a size range of 2.6 to 3.0 cm TL (mean = 2.86 ± 0.13 cm SD) at 15°+ 2° C. A linear model provided the best fit of the size-age data with a regression formula of y = 23.3 x – 9.9, r^2^=0.92, p < 0.001, Figure S1. Because of the strong relationship between size and post-hatch age (r^2^ = 0.92) of the midshipman we are able to estimate the age of fish used in this study that were not included in the growth analysis, but were used in the AEBR experiments, by measuring the TL of the fish.
